# Ischemic preconditioning induces neuroprotection cause by a transient global ischemia via maintaining the expression of p63

**DOI:** 10.1186/2197-425X-3-S1-A771

**Published:** 2015-10-01

**Authors:** JH Cho, CW Park, TG Ohk, MC Shin, YS Kim, MH Won

**Affiliations:** Kangwon National University, Emergency Medicine, Chuncheonsi, Republic of Korea; Kangwon National University, Neurobiology, Chuncheonsi, Republic of Korea

## Introduction

p63 is a transcription factor of p53 gene family, which are involved in development, differentiation and cell response to stress; however, their roles in ischemic preconditioning (IPC) in the brain are not clear.

## Objectives

In the present study, we investigated the effect of IPC on p63 immunoreactivity caused by transient cerebral ischemia, which was induced by 5 min of transient ischemia, in gerbils, and IPC was induced by subjecting the gerbils to 2 min of ischemia followed by 1 day of recovery.

## Methods

The animals were randomly assigned to 4 groups (sham-operated-group, ischemia-operated-group, IPC plus (+)-sham-operated-group and IPC+ischemia-operated-group).

## Results

The number of viable neurons in the stratum pyramidale of the hippocampal CA1 region (CA1) was significantly increased by IPC+ischemia-operated-group compared with that in the ischemia-operated-group 5 days after ischemic insult. We found that strong p63 immunoreactivity was detected in the CA1 pyramidal neurons in the sham-operated-group, and the immunoreactivity was decreased with time after ischemia-reperfusion. In addition, strong p63 immunoreactivity was newly expressed in the microglial cells of the CA1 region from 2 days after ischemia-reperfusion. In all the IPC+sham-operated-group, p63 immunoreactivity in the CA1 pyramidal neurons was similar to that in the sham-operated-group, and the immunoreactivity was well maintained in the IPC+ischemia-operated-groups after cerebral ischemia.

## Conclusions

In brief, our present findings showed that IPC dramatically protected the reduction of p63 immunoreactivity in the pyramidal neurons of the CA1 region after ischemia-reperfusion, and this result suggests that the expression of p63 may be necessary for the neurons to survive after transient cerebral ischemia.

